# Cell Death Effects Induced by Sulforaphane and Allyl Isothiocyanate on P-Glycoprotein Positive and Negative Variants in L1210 Cells

**DOI:** 10.3390/molecules25092093

**Published:** 2020-04-30

**Authors:** Szilvia Kontar, Denisa Imrichova, Anna Bertova, Katarina Mackova, Alexandra Poturnayova, Zdena Sulova, Albert Breier

**Affiliations:** 1 Institute of Molecular Physiology and Genetics, Centre of Biosciences, Slovak Academy of Sciences, Dúbravská cesta 9, 84005 Bratislava, Slovakia; szilvia.kontar@savba.sk (S.K.); anna.bertova@savba.sk (A.B.); katarina.mackova@savba.sk (K.M.); alexandra.poturnayova@savba.sk (A.P.); 2 Institute of Biochemistry and Microbiology, Faculty of Chemical and Food Technology, Slovak University of Technology in Bratislava, Radlinského 9, 81237 Bratislava, Slovakia

**Keywords:** P-glycoprotein/ABCB1, multidrug resistance, sulforaphane, allyl isothiocyanate, autophagy, LC3B, NF-κB pathways, apoptosis

## Abstract

Variants of L1210 leukemia cells-namely, parental P-glycoprotein-negative S cells and R and T cells expressing P-glycoprotein, due to selection with vincristine and transfection with the human p-glycoprotein gene, respectively-were used. The responses of these cell variants to two naturally occurring isothiocyanates-sulforaphane (SFN, from cruciferous vegetables) and allyl isothiocyanate (AITC, from mustard, radish, horseradish and wasabi)-were studied. We obtained conflicting results for the cell death effects induced by isothiocyanates, as measured by i. cell counting, which showed inhibited proliferation, and ii. cell metabolic activity via an MTS assay, which showed an increased MTS signal. These results indicated the hyperactivation of cell metabolism induced by treatment with isothiocyanates. In more detailed study, we found that, depending on the cell variants and the isothiocyanate used in treatment, apoptosis and necrosis (detected by annexin-V cells and propidium iodide staining), as well as autophagy (detected with monodansylcadaverine), were involved in cell death. We also determined the cell levels/expression of Bcl-2 and Bax as representative anti- and pro-apoptotic proteins of the Bcl-2 family, the cell levels/expression of members of the canonical and noncanonical NF-κB pathways, and the cell levels of 16 and 18 kDa fragments of LC3B protein as markers of autophagy.

## 1. Introduction

Isothiocyanates (ITCs) are a group of substances occurring in nature (allyl isothiocyanate, sulforaphane, etc.) or as chemical synthetic products (fluorescein isothiocyanate, *p*-bromophenyl isothiocyanate, etc.) for which a wide range of biological effects are known [1,2]. Their effects can be derived from the reactivity of the ITC group (−NCS) and the physicochemical properties (lipophilicity, shape, size and rigidity) of the rest of the molecule. While the former predetermines the ability of ITCs to react with the functional groups of either small biochemical molecules or biopolymers [3], the latter is responsible for their bioavailability in different compartments of cells and tissues [4]. NCS groups are accessible for attack with nucleophilic functional groups with a free electron pair and a partial negative charge. In biological materials, the most common reactive partners of ITCs are −SH, −OH and −NH_2_ groups [3]. When expressing the affinity of a primary amino group for reaction with ITC in arbitrary units (and setting it equal to 1), the affinity of the hydroxyl group is 0.2, and the affinity of the sulfhydryl group ranges within the interval 1000–1,000,000 [3,5,6]. However, the reaction of ITCs with −NH_2_ groups results in stable *N,N’*-disubstituted thiourea derivatives, as opposed to reactions with the −SH or −OH groups, which provide S- or O-esters of thiocarbamic acid with poor stability [3,7,8].

Despite the assumption that ITCs as a group-specific reagent should more or less stochastically interact with nucleophilic groups of proteins, it has been shown in the past that specific labeling of the essential functional groups of the active site of the enzymes may occur. An example is selective modification of the essential thiol group of d-glyceraldehyde-3-phosphate dehydrogenase by alkyl-, aryl- and aralkyl-ITCs [6] or specific labeling of the Na^+^/K^+^-ATPase binding site with fluorescein isothiocyanate on the essential −SH [7,8] or −NH_2_ groups [9].

The biological effects of ITCs resulting from their chemical properties have been studied intensively over the previous four decades of the last century. We are currently seeing a renewal of interest, particularly regarding natural ITCs occurring in plants, due to the accumulation of data on the chemopreventive effects of ITC against chronic diseases such as neoplastic, cardiovascular, neurodegenerative and metabolic diseases [10]. However, the same authors have pointed out the genotoxicity of plant ITCs, which can either result in the specific intervention of neoplastically transformed cells to eliminate them or damage normal cells by causing mutations in their genes. In addition to these effects, ITCs, by chemical modification of bio(macro)molecules, can modulate the transcription/function of a broad spectrum of enzymes/proteins [11]. It seems evident that ITCs preferentially target transcriptionally highly active cells with elevated metabolic turnover and a high incidence of proliferation, which are also characteristics of neoplastically transformed cells [12]. Therefore, it is not surprising that plant ITCs attack cells in cell lines derived from neoplasia of various tissues and reduce their survival and proliferation levels, as reviewed in [13].

**Figure 1:**
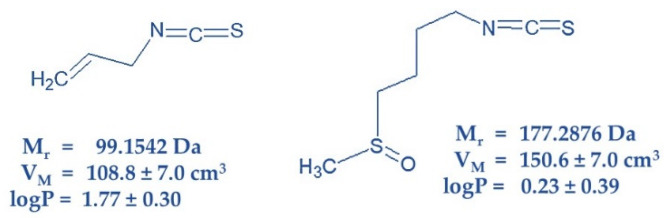
Structure of AITC (left) and SFN (right) characterized by physico-chemical constants Mr-relative molar mass; VM-molar volume and logP-logarithm of partition coefficient in two-phase mixture form by water and n-octanol. Structures were drawn, and physico-chemical constants were calculated using ACD/ChemSketch for academic and personal use (Advanced Chemistry Development, Inc., Toronto, ON, Canada).

Available sources suggest that the amount of different ITC in its vegetable producers vary from 60 to 400 mg/100 g wet weight [14]. However, this content could be reduced by improper storage. The cytotoxic and carcinostatic efficacy of sulforaphane (SFN, present in cruciferous vegetables) and allyl isothiocyanate (AITC, responsible for the pungent taste of mustard, radish, horseradish and wasabi) was established in 1968 via in vitro experiments with HeLa cells [15]. The anticancer effectiveness of both ITCs was confirmed in later works [16,17]. Structures and physico-chemical properties of both ITCs are documented on Figure 1.

Both isothiocyanates are found in plants in the form of stored glucosynolates, which are poorly bioavailable [18]. In the diet, after its mechanical disruption, bioavailable isothiocyanates are released by the enzyme myrosinase. Therefore, not only the content of glucosynolates, but also the content of active myrosinase and the method of processing plant food are important factors for the administration of ITC in organisms to induce their beneficial effects. Malignant cells can develop resistance to a wide group of structurally unrelated substances with different mechanisms of anticancer action-multidrug resistance (MDR) [19]. The overexpression of a drug efflux pump of the plasma membrane P-glycoprotein (P-gp, ABCB1 member of ABC transporter gene family) represents the most frequently observed molecular cause of MDR [20,21]. Identifying substances that suppress malignant cell growth and are not P-gp substrates and/or P-gp inducers represents an important aim in medicinal chemistry research [22]. For this purpose, the effectiveness of substances on P-gp-negative and P-gp-positive cell variants must be compared.

In 2010 [23], we established the following panel of mouse leukemia cells suitable for the study of substance effectiveness in relation to P-gp drug efflux activity: i. drug-sensitive P-gp-negative murine leukemia cells l-1210 (S cells); ii. drug-resistant P-gp-positive cells obtained by adaptation of S cells to vincristine (R cells); iii. drug-resistant P-gp-positive cells obtained by S cell transfection with a human gene encoding P-gp (T cells). We successfully used these three variants to characterize the cytotoxic effects of triorgano-stannane derivatives [24] and phenanthroquinolizidine derivatives [25] in relation to the presence of P-gp.

In this paper, we compared the cytotoxic effects of SFN and AITC on S, R, and T variants of L1210 cells. Another aim of our work was to characterize the molecular mechanism of the cytotoxic effects of both ITCs on S, R and T cells.

## 2. Results

### 2.1. Cell Death Effects of SFN and AITC on S, R and T Cells

Two methodologies of cell viability detection were used in our experiments: i. counting the absolute number of viable cells based on the detection of single cell viability using the CASY Model TT Cell Counter; ii. measuring metabolic activity by monitoring the NADH/NADPH-dependent reduction of tetrazolium dye to formazan using the MTS assay.

**Figure 2:**
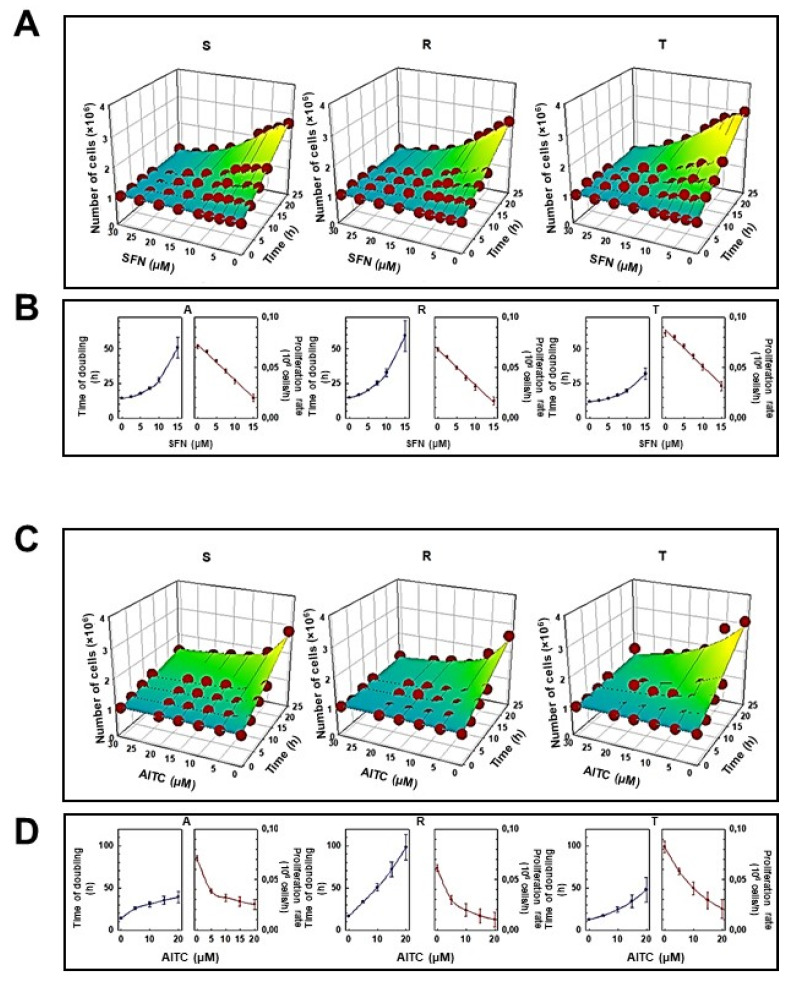
Effect of SFN (panel **A**,**B**) and AITC (panel **C**,**D**) on the proliferation of S, R and T cells. Experimental data (Numbers of viable cells from three independent experiments, S.E.M. values never exceed 10%) were fitted according to Equation by nonlinear regression and were plotted as a function of culturing time period (0–24 h) and concentration of either SFN (panel **A**) or AITC (panel **C**) in three-dimensional mesh plots. Values of doubling times and proliferation rate together with the corresponding standard errors were computed from Equation and are plotted as a function of either SFN (Panel **B**) or AITC (Panel **D**) concentration.

The effect of SFN and AITC on the proliferation of S, R, and T cells measured by the former procedure is summarized in Figure 2. These agents suppressed cell proliferation in a concentration-dependent manner over a concentration range of 5–20 μM. At the highest concentration of both ITCs, the proliferation of S, R and T cells was practically stopped, and the number of viable cells during culturing was similar to the inoculum value (10^6^ cells, Figure 2A,C). These panels also revealed a satisfactory match between the experimental data and those calculated by nonlinear regression according to Equation. Such a match makes it possible to reliably determine the cell doubling time and the proliferation rate, which are summarized in Figure 2 (panels B and D), and the IC_50_ value (Table 1).

The values of IC_50_ (Table 1) indicate that during the 24 h experiment there is no order of magnitude difference in the response of S, R and T cells to the presence of both isothiocyanates used. Thus, the changes in IC_50_ values cannot be considered as changes in the sensitivity of the cells that will considerably alter cell response to ITCs.

**Figure 3:**
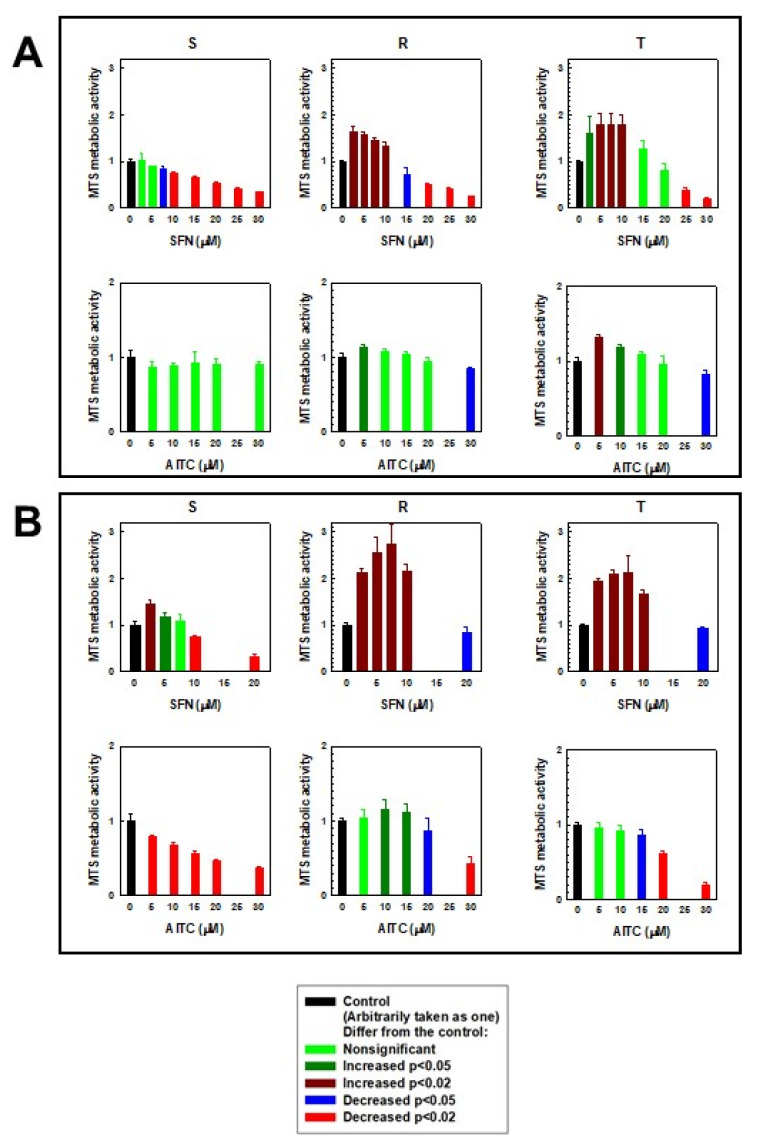
Effects of SFN and AITC on the metabolic activity of S, R and T cells measured by an MTS assay after 24 h (panel **A**) and 48 h (panel **B**). Experimental data represent the means ± S.E.M. from five independent experiments. The significance of increases or decreases is given in the legend.

Another situation occurred when the effect of SFN and AITC on S, R and T cells was monitored by the MTS test (Figure 3). The formazan signal in R and T cells after 24 h of culture in the presence of SFN increased at concentrations less than 10 μM (R cells) or 15 μM (T cells). Similarly, we observed an increase (but more pronounced) in the absorbance of formazan in R and T cells after 48 h of culture in the presence of SFN, where the limiting concentration was 20 μM. In S cells, a significant increase in formazan absorbance was observed only after 48 h of culture in the presence of 2.5 and 5.0 µM SFN. At concentrations of SFN higher than the limits described above, we detected significant decreases in formazan absorbance in all three variants of L1210 cells. A slight increase in formazan absorbance (as in the previous case) was also observed in R and T cells when AITC was administered to the culture medium (Figure 3). In contrast, the absorbance of formazan decreased monotonically in relation to increasing concentrations of AITC in S cells.

### 2.2. Detection of SFN- and AITC-Induced Cell Death Mode of S, R and T Cells Using Double Staining with Annexin-V and Propidium Iodide

**Figure 4:**
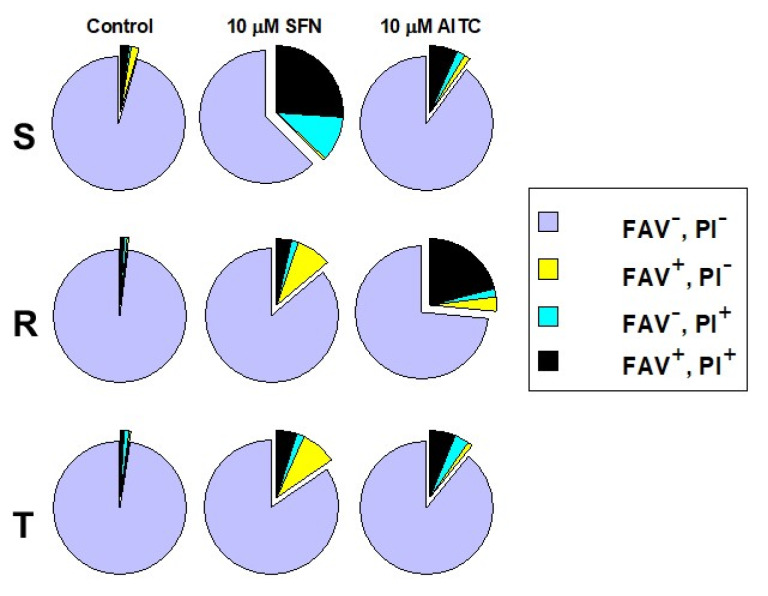
Determination of cell viability by FAV/PI assay. The cells were incubated for 48 h in the absence (control) or presence of 10 µM of either SFN or AITC prior to the measurements. Legends: FAV^−^, PI^−^–viable cells; FAV^+^, PI^−^–apoptotic cells; FAV^–^, PI^+^–necrotic cells and FAV^+^, PI^+^–cells in late state of death. Data in the pie charts are representative of three independent measurements. Respective FACS dot plots are documented in Supplementary files (Figure S1).

Mode of cell death after 48 h of culturing of S, R and T cells in the presence of SFN and AITC (10 μM) was estimated by using cell double staining with fluorescein isothiocyanate labeled annexin-V (FAV, a marker of cell apoptosis) and propidium iodide (PI, marker of cell necrosis). The results are summarized in Figure 4. P-gp-negative S cells are more sensitive to SFN than P-gp-positive variants. This may be documented by an increase in the cell proportion that was stained with either PI alone or PI together with FAV. While the former staining indicates the initiation of necrosis, the latter staining is attributed to the late cell death stage, to which cells may arrive via both mechanisms of cell death. Thus, we consider necrosis to be a prevalent mode of death in S cells under SFN treatment.

SFN induced an increase in the proportion of cells stained with FAV alone in R and T cells in contrast to S cells, for which this staining indicated that apoptosis progression was not observed. Additionally, an elevation in the cell proportion stained by both FAV and PI was obtained (Figure 4). Therefore, apoptosis is involved in cell death induced by SFN in R and T cells.

AITC (10 μM, after 48 h culturing) affected R cells most potently from all L1210 cell variants according to the increase in the proportion of cells stained with both FAV and PI (Figure 4). S and T cells responded to this treatment similarly. However, almost all R and T cells (97%) died after treatment with massive concentrations of ITC (30 μM), while 76% of S cells were able to survive in this condition (Supplementary data, Table S1). When SFN was applied at this concentration, there were no viable cells (i.e., negative on FAV and PI staining) after 48 h incubation (data not shown).

### 2.3. Expression of Proteins Active in Apoptosis in S, R and T Cells Treated with either SFN or AITC

**Figure 5:**
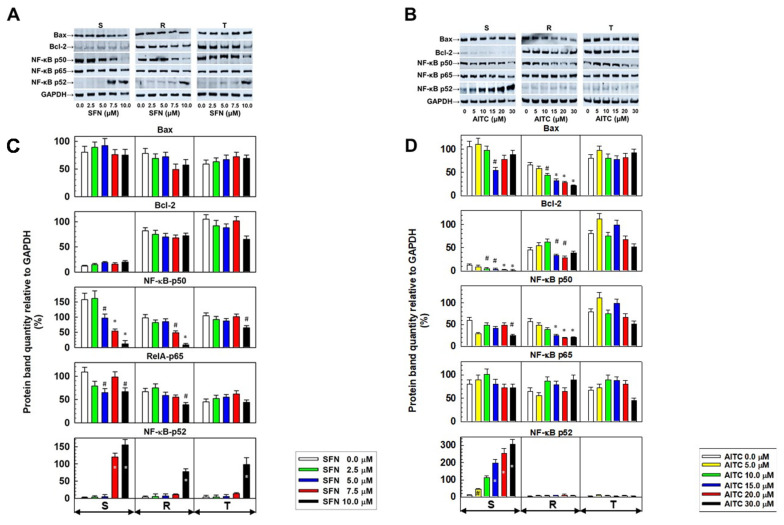
Expression of proteins that are active in apoptosis (Bcl–2, Bax, and the members of NF-κB pathways: p50, P52 and p65) in S, R and T cells after 24 h of incubation in medium containing given concentrations of SFN or AITC. Panels **A** and **B**: Detection of proteins by Western blotting. GAPDH was used as an internal control. Data are representative of three independent measurements. Panels **C** and **D**: Densitometric quantification of protein bands from Western blots. Quantities are expressed as the mean ± S.E.M. of three independent measurements of data relative to the GAPDH signal. Data differ from values obtained in cells that were not incubated in the absence of either SFN or AITC at the levels: # – *p* < 0.05; * – *p* < 0.01.

Both P-gp-positive L1210 cell variants contained higher levels of antiapoptotic Bcl-2 protein than P-gp-negative S cells (Figure 5). We also verified this result at the transcript level (Supplementary files, Figure S2). SFN caused a slight increase in Bcl-2 protein levels in S cells, but the change was not significant. In contrast, Bcl-2 levels decreased significantly in S cells depending on the concentration of AITC. A slight decrease in this protein was also observed in R cells at higher AITC concentrations (Figure 5). Changes in the Bcl-2 gene transcript in relation to SFN or AITC treatment were not present (Supplementary files Figure S3). Almost identical levels of pro-apoptotic Bax proteins were observed in S, R and T cells (Figure 5). While SFN did not induce significant changes in this protein in all three cell variants, in R cells, the levels of Bax decreased depending on AITC concentration. Consistent with the Western blotting data, we also detected similar levels of Bax gene transcripts in all three cell variants, and these expression levels were independent of treatment with SFN or AITC (Supplementary files Figure S2).

The regulatory pathways NF-κB (canonical and non-canonical with p50 and p52 as transcription factors) exhibit anti-apoptotic activity [26,27]. Therefore, we studied the level of protein expression of these pathways in S, R and T cells depending on the treatment with SFN and AITC (Figure 5). After treatment with SFN, we observed a decrease in the p50 protein level of the canonical NF-κB pathway, which was accompanied by the upregulation of the noncanonical p52 pathway member (Figure 5). This was mostly pronounced in S cells, but statistically significant changes were also obtained for R and T cells at higher concentrations. The levels of Rel A (NF-κB p65 protein), the dimerization partner of the p50 protein, seemed less dependent on SFN treatment. AITC induced a decrease in p50 to a lesser extent than SFN. However, treatment with AITC induced an increase in the p52 levels in S cells in a concentration-dependent manner.

We also checked the expression of p50, P52 and p65 as members of both NF-κB pathways in S, R and T cells in relation to either SFN or AITC treatment at the level of their gene transcripts. There was no significant change in the levels of the respective mRNAs in relation to treatment with SFN and AITC (Supplementary data Figure S2). However, we detected an increase in the level of RelB transcript (which protein product is considered to be a member of the noncanonical NF-κB pathway but a dimerization partner of both p50 and p52 proteins [27]) in S cells when treated with both ITCs. The expression of this transcript appears to be rather independent or downregulated in R and T cells after treatment with SFN and AITC.

### 2.4. Effect of SFN and AITC on the Cell Cycle of S, R and T Cells

**Figure 6:**
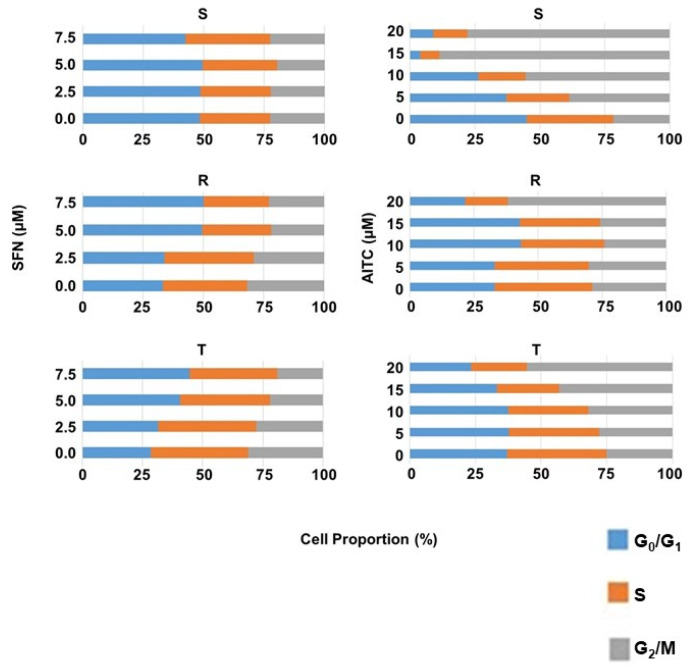
Summarization of the cell cycle phases (G0/G1, S and G2/M) of S, R and T cells after culture in the absence or presence of SFN for 8 h or AITC for 12 h at the given concentrations. Data are representative of three independent measurements, and the respective FACS histograms are documented in the Supplementary Files (Figure S3). AITC caused the greatest CC changes in S cells, which caused a concentration-dependent increase in the cell fraction in G2/M (Figure 6). We also registered an increase in the proportion of cells in the G2/M phase in R and T cells, but this was less pronounced than in S cells.

The effect of SFN and AITC on the cell cycle (CC) was examined by determining the cellular DNA content of S, R, and T cells after 48 h of culture in the absence or presence of either SFN (at 2.5, 5.0 and 7.5 μM) or AITC (at 5, 10, 15 and 20 μM) in a flow cytometer (Figure 6). Treatment of R and T cells with SFN (particularly at concentrations of 5.0 and 7.5 µM) caused an increase in the cell fraction in the G0/G1 phase of CC, which was counterbalanced by reducing the proportion of cells in the other CC phases, i.e., S and G2/M. In contrast to R and T cells, the proportion of S cells in the different phases of CC was practically unchanged after such treatment with SFN.

### 2.5. Effect of SFN and AITC Treatment on the Molecular Forms of LC3B as Autophagy Markers in S, R and T Cells

The molecular forms of LC3B protein include either cytosolic LC3B1 (18 kDa) or autophagosomal membrane LC3B2 (16 kDa) originating from specific cleavage of minimally detected pro-LC3B with Mr–30 kDa are generally accepted as autophagy markers [28,29]. In mouse, pro-LC3B is a product of the Map1lc3b gene (Gene ID 67443 in *NCBI RefSeq genomes*). Therefore, we must check whether SFN or AITC treatment induces changes in the cellular levels of these two molecular forms.

**Figure 7:**
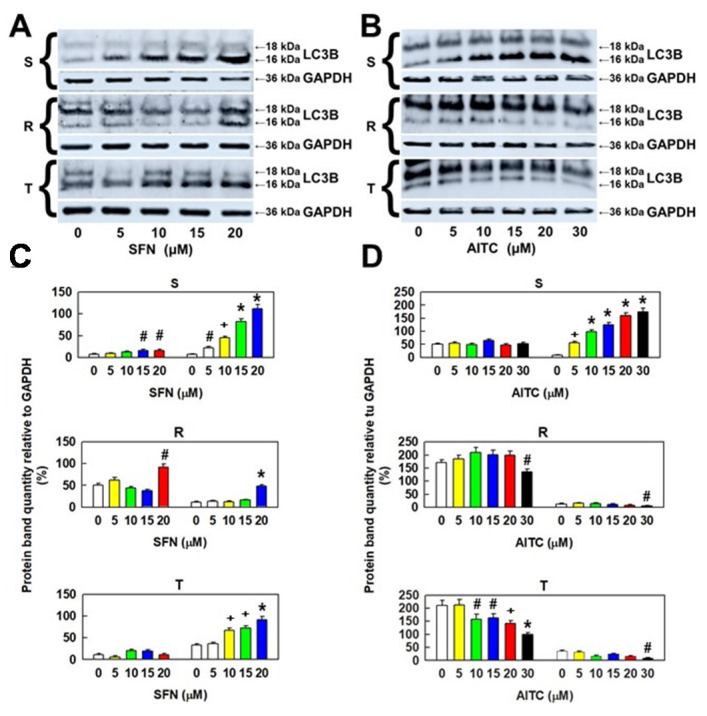
Cellular levels of LC3B protein variants in S, R and T cells after incubation in medium containing different concentrations of SFN or AITC for 24 h. Panels **A** and **B**: Detection of proteins by Western blotting. GAPDH was used as an internal control. Data are representative of three independent measurements. Panels **C** and **D**: Densitometric quantification of protein bands from the Western blots. Quantities are expressed as the mean ± S.E.M. from three independent measurements of data in relation to the GAPDH signal. Data differ from values obtained in cells that were not incubated in the absence of either SFN or AITC at the following levels: # – *p* < 0.05; + – *p* < 0.02; * – *p* < 0.01.

While the 16 kDa variant of LC3B was considerably increased in S cells after treatment with SFN (in a concentration-dependent manner), the other 18 kDa variant was less upregulated (Figure 7). The membrane 16 kDa variant LC3B was more upregulated in T cells than in R cells but less upregulated in S cells. However, there was a significant increase in the 18 kDa cytosolic variant of LC3B in R cells after treatment with 20 µM SFN, which was absent in T cells. Similarly, as with SFN, AITC also induced a concentration-dependent increase in the levels of the smaller variant of LC3B in S cells, but without any change in the larger variant levels (Figure 7). We observed a decrease, rather than an increase, in the levels of both proteolytic products of the pro-LC3B protein in P-gp-positive variants of L1210 cells (R and T).

These results suggest that autophagy associated with translation of the pro-LC3B protein and its proteolytic processing to 16- and 18-kDa protein variants plays a role in the effect of SFN and AITC on the cell variants S, R, and T.

### 2.6. Detection of Lysosomal Compartment in S, R and T Cells after Treatment with SFN and AITC

Autophagic vesicle formation can be observed by staining cells with monodansyl cadaverine (MDC) [30]. We also used this procedure to monitor autophagy in S, R and T cells after incubation with SFN and AITC. In addition, we used wheat germ agglutinin (WGA) conjugated with Alexa Fluor™ 647 to visualize the plasma membrane. This lectin massively marks the surface of S, R and T cells [31,32]. It is known that fluorescent indicators can be a substrate of P-gp and thus can interfere with the assay [33,34]. In a previous work, we showed that inhibition of P-gp with tariquidar (TQR) allows the labeling of mitochondria with JC–1 in P-gp-positive R and T cells to the same extent as in S cells [35]. Therefore, we verified whether MDC application required blocking P-gp activity with TQR.

**Figure 8:**
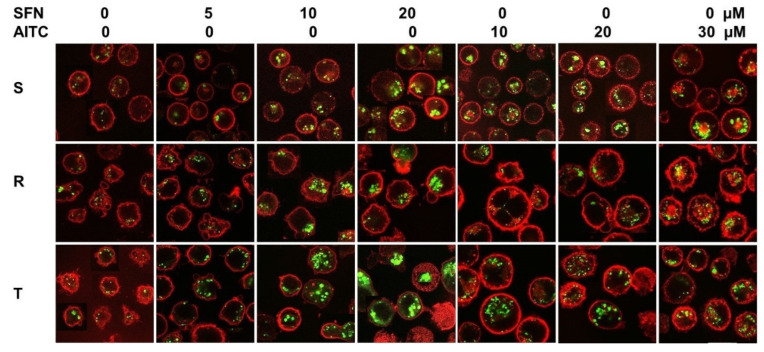
Visualization of autophagic vesicles in S, R and T cells using MDC. Cells were stained by MDC in the presence of 500 nM TQR. Cells after 24 h of incubation in medium containing SFN and AITC at the given concentrations were used in the experiment. Data are representative of three independent experiments.

While we recorded MDC fluorescence in S cells even in the absence of TQR, in P-gp-positive R cells in the absence of TQR, its signal was weak (Supplementary files Figure S4). However, when TQR (500 nM) was used, MDC fluorescence in R cells reached values comparable to those in S cells. In a previous work [35], we verified that such a concentration of TQR would not affect the viability of S, R and T cells. Therefore, in further experiments of MDC staining, we added TQR to each sample. Under these conditions, we detected basal fluorescence of MDC in cells without induction with ITCs. Relatively small intracellular structures are labeled (Figure 8). Both ITCs concentration-dependently induced elevation of MDC fluorescence in the intracellular space, and more robust structures were labeled.

**Figure 9:**
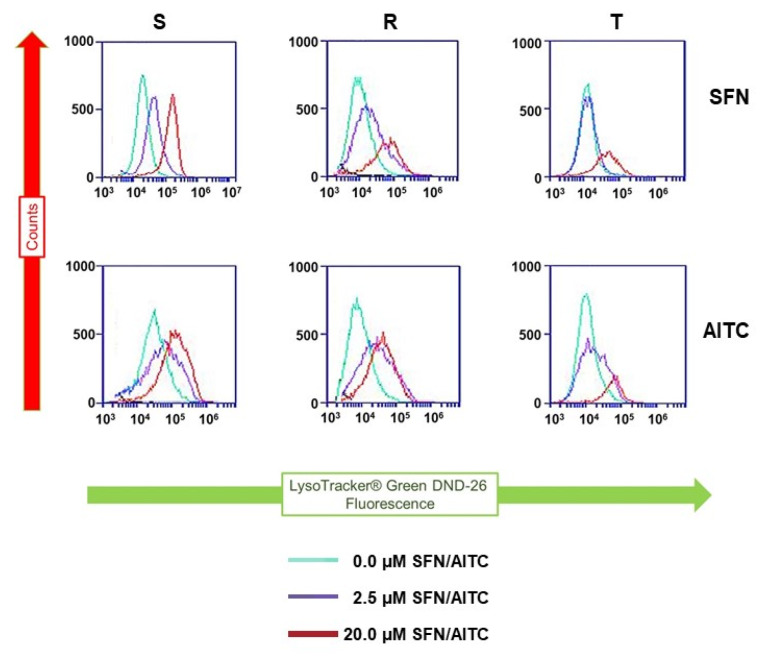
L1210 cell variants (S, R and T) were treated with SFN and AITC (at given concentrations) for 24 h and then stained with LTG. In the past, LTG has been shown to be useful for detecting the efflux activity of BCRP [37], which is an ABCG2 member of the ABC transporter family and is known to be associated with MDR. Although LTG has not yet been described as a P-gp substrate, we applied the P-gp inhibitor TQR at a concentration of 500 nM to maintain the conditions of the experiment documented in Figure 8. Data are representative of three independent measurements.

Cells in which autophagy was induced by different treatments have elevated cellular levels of cleaved LC3B variants that were associated with an increase in cell staining with the lysosome-specific dye LysoTracker^®^ green DND-26 (LTG) [36]. This fact we used for further monitoring of the possible pro-autophagic effect of both ITCs on S, R and T cells. Incubation of S, R and T cells in medium containing either SFN or AITC increased cell staining with this indicator as measured by flow cytometry (Figure 9).

All of these results indicate that after treatment with SFN and AITC, S, R and T cells become positive for the specific detection of autophagy with fluorescent indicators.

## 3. Discussion

In monitoring the cytotoxic effect of the ITCs used, we obtained contradictory results upon comparing the effect of substances by cell counting using a cell counter (Figure 2) and determining the effect of substances using an MTS cell proliferation assay (Figure 3). While the number of cells with increasing concentrations of ITCs decreased monotonically, the MTS signal was increased at lower concentrations of ITCs (especially SFN). This is an unusual phenomenon because in most cases, a clear correlation between the cell counting results and the colorimetric MTS assay is observed. The MTS assay is often referred to as a “Cell proliferation assay”. This information is also available in the manufacturer’s protocols. However, this assay is based on the cellular reduction of the tetrazolium dye. Succinate, NADH and NADPH are required for such a reduction [38]. The tetrazolium dye reduction occurs not only with mitochondrial involvement but also as a result of cytoplasmic enzyme activity and on non-mitochondrial membranes, including the endosome/lysosome compartment and plasma membrane [39]. Cells can respond to stress conditions by stimulating their metabolism, and this metabolic hyperactivation can provide resources to cope with stress. Metabolic hyperactivation of cells can elevate the cellular content of succinate, NADH and NADPH and therefore may represent a limit in the application of the MTS (or MTT) assay for the detection of cell viability. This feature was highlighted by Rai et al. [40] in the case of metabolic hyperactivation of cells caused by radiation exposure. Therefore, our contradictory results described above suggest that the effects of SFN and, to a lesser extent, AITC are due to metabolic hyperactivation of particularly P-gp-positive R and T cells.

It appears that cell death induced by SFN and AITC is controlled by a variety of mechanisms, including apoptosis combined with CC arrest (reviewed in [41]). However, on the one hand, autophagy [42], and on the other hand, the induction of an imbalance in the formation and quenching of reactive oxygen species that leads to necrosis [43], could be involved in cell damage. It must be considered that SFN-mediated MAPK signaling could play an important role in this process. The latter idea is deduced from fact that SFN can induce reactive species of oxygen, and activates extracellular signal-regulated kinases which in turn facilitates autophagy [44]. Therefore, these and other mechanisms (not mentioned above) could result in the effect of ITCs leading to cell death. Their synergy will contribute to impairing cellular functions and the subsequent elimination of damaged cells. This suggests that the cell damage processes involved in the death of particular cells will depend on the type of cells and their metabolic state, which corresponds to the specific profile of the expressed enzymes or regulatory proteins. Therefore, it is not surprising that different cell lines may respond differently to treatment with ITCs under different culture conditions. Our results show an example of the difference in effect depending on the cell variant and their specific features. After treatment of the P-gp-negative cell variant S with SFN, we did not observe any increase in the proportion of cells undergoing apoptosis (i.e., labeled exclusively with FAV); in contrast to P-gp-positive cells R and T, in which SFN treatment induced real elevation of apoptotic cell numbers (Figure 4). However, SFN affected S cells more effectively than R and T cells but via necrosis. This effect cannot be caused by the efflux activity of P-gp present in R and T cells since SFN does not alter the transport activity of this ABC transporter as measured by cellular accumulation or efflux of doxorubicin and rhodamine 123 as P-gp fluorescent substrates [45]. This result suggests that SFN is not an effective substrate and/or inhibitor of P-gp. AITC induced cell death in P-gp-positive R cells more effectively than in P-gp-negative S cells. Interestingly, after treatment of cells with 10 μΜ AITC, similar responses of S and T cells were registered (Figure 4). In our previous papers, when the cell death effects of various substances (which are not P-gp substrates) were examined, similar responses of P-gp-positive R and T cells and different responses of S cells were always obtained [22,23,43]. However, the data in Table S1 (in Supplementary files) indicated a similar sensitivity of T and R cells to AITC at higher concentrations (30 μΜ) and the considerably lower sensitivity of S cells. The predominant proportion of R and T cells after treatment with this higher concentration of AITC was found in the late phase of death (i.e., cells labeled with both FAV and PI). Thus, differences in susceptibility to SFN and AITC exist between P-gp-negative S cells and P-gp-positive R and T cells, and, moreover, the pathways by which these cell variants reach cell death, differ.

The anti-apoptotic proto-oncogene Bcl-2 is implicated in mechanisms that prevent neoplastically transformed cells from entering the process of apoptosis, i.e., apoptosis, to undergo self-elimination [46,47]. Therefore, the anti-apoptotic action of Bcl-2 contributes to the pathological phenotype of neoplastic cells, which allows cancer progression [48]. In leukemic patients, co-expression of both P-gp and Bcl-2 can be found, indicating impaired therapeutic prognosis [49,50]. Our P-gp-positive R and T cells expressed much more Bcl-2 at both the mRNA (Figure S2, in Supplementary files) and protein (Figure 5) levels. While after treatment with SFN, we did not detect significant changes in the Bcl-2 protein in S, R and T cells, treatment with AITC caused a significant decrease in the cellular content of this protein in S and R cells. The pro- apoptotic member of the Bcl-2 family, Bax protein, was expressed approximately equally in S, R and T cells. Additionally, in this case, treatment with SFN did not induce changes in the cell content of Bax, but treatment with AITC reduced its level in R cells. However, these changes did not induce a marked acceleration of apoptosis as measured with FAV and PI (Figure 4).

Several lines of evidence suggest that ITCs could be beneficial as preventive agents against the initiation and progression of early forms of neoplasia by suppressing NF-κB signaling pathways. This may be documented in the results of the following two papers: Xu et al. [51], that the inhibition of IKKα and IKKβ phosphorylation by SFN and phenethyl isothiocyanate and subsequent blocking of downstream processes leads to a reduction in NF-κB-induced proteins transcription as proapoptotic stimuli; Wagner et al. [52], that AITC reduced p65 protein levels in nuclei in lipopolysaccharide-stimulated murine RAW264.7 macrophages. Inhibition of the canonical or noncanonical NF-κB pathways with either RNA interference or transfection with a gene encoding a stable form of IκB (NF-κB pathway inhibitor) enhanced the sensitivity of cells to apoptosis inducers [53]. Therefore, we further focused on the determination of the expression of the canonical (Nfkb1 and RelA) and noncanonical (Nfkb2 and RelB) genes of the NF-κB pathway (Figure S2 in Supplementary files) and proteins of either p50 (Nfkb1 gene product) and p65 (RelA gene product) of the canonical pathway or p52 (Nfkb2 gene product) of the noncanonical pathway (Figure 5). Our results suggest that especially in S cells, SFN or AITC treatments induce a transition from the canonical to noncanonical NF-κB pathway. The results for R and T cells appear less pronounced. In his review, Sun [54] pointed out the following differences in the characteristics and performance of NF-κB pathway variants:

- The canonical pathway is initiated by many signals, including those that mediate innate and adaptive immune receptors. This signaling includes degradation of the NF-κB inhibitor, proteolytic cleavage of the p105 precursor to functional p50, generation of the transcriptionally active p65/p50 heterodimer, translocation to the nucleus and rapid but transient expression of target genes.
- The noncanonical NF-κB pathway is induced by sets of tumor necrosis factor receptors, followed by proteolysis of precursor p100 to functional p52, which dimerizes with RelB (a *RelB* gene product). This dimer in the nuclei persistently but slowly induces the transcription of genes regulated by this cascade.

While these ideas allow several deductions about the functions of such changes in the expression of members of the alternative NF-κB pathways after the treatment of cells with SFN and AITC, further targeted research will be needed to fully understand this phenomenon.

Studying the effects of SFN and AITC on CC progression (Figure 6) in S, R and T cells revealed antipodal results: i. SFN induced a more pronounced effect on R and T cells than on S cells, while AITC induced the highest effect on S cells, a less pronounced effect on T cells and only a negligible effect on R cells; ii. SFN induced cell arrest in the G0/G1 phase and AITC in the G2/M phase of CC. Consistent with our results, CC arrest in the G0/G1 phase and the G2/M phase has already been described for human bladder cancer T24 cells after treatment with SFN [55] and for human prostate cancer cells (PC-3-androgen-independent and LNCaP-androgen-dependent) [56], respectively. According to structure (Figure 1), the −NCS group of both ITCs should exert similar reactivity with nucleophilic groups because both are alkyl-ITCs and the −NCS group is divided from the rest of the molecule by one (AITC) or four (SFN) −CH_2_− links of the hydrocarbon chain [3,5,6].

Therefore, the difference in the mode of action and efficacy of SFN or AITC on S, R and T cells should not be based on the reactivity of −NCS groups but rather on the physico-chemical properties of residual parts of their molecules. These two ITCs differ from each other in i. lipophilicity, since AITC achieves a higher partition coefficient in a two-phase water: n-octanol mixture; ii. the size of molecules deduced from molar volume, which is higher for SFN than for AITC; iii. the presence of a terminal double C=C bond on the AITC molecule and the presence of a −SO− linker on the SFN molecule (Figure 1). All the above differences may be responsible for their accumulation in different cell compartments and for differences in the response of cells after treatment with them.

Cells after ITC administration either appear to stop CC progression (SFN-treated R and T cells or AITC-treated S and T cells) or undergo cell death (SFN-treated S cells; AITC-treated R cells). Cells with arrested CC progression appear to repair ITC-induced damage, which can lead to survival in the event of successful repair or death when repair fails. During the period in which the cells are arrested in the G0/G1 or G2/M phase of the CC, due to the toxic stress induced by SFN or AITC, autophagy may be activated to provide the necessary chemicals and energy for repair [57]. Autophagy can recycle sources (amino acids, sugars, and nucleotides) from endogenous stores in the cell. This recycling of cell materials can help cells eliminate damaged biomacromolecules, acquire material and energy resources to synthesize new ones and, thus, adapt and survive under adverse stress conditions [58]. Therefore, we determined the cellular levels of both 16 kDa and 18 kDa proteolytic products of the LC3 protein (which are recommended autophagy markers [59]) by Western blotting (Figure 7). Measurable amounts of these small peptides have been shown to be present in S, R and T cells, and their amount depends on the concentration of SFN or AITC used for cell treatment.

Another cell marker useful for the visualization of autophagic vesicles is MDC, a dye that emits blue fluorescence and is thought to preferentially accumulate in autophagic vesicles but not in early and late endosomes [59]. MDC accumulation in autophagic vesicles is exclusively due to a combination of ion trapping and specific interactions with vesicle membrane lipids [60]. This marker appears to be transportable by the efflux activity of P-gp, since it accumulates substantially less in R and T cells than in S cells, and its retention in P-gp-positive cells can be achieved by the presence of the noncompetitive high-affinity P-gp inhibitor TQR (Figure S4 in Supplementary files). Thus, MDC is ranked among the fluorescent indicators (such as calcein/AM, Fluo–3/AM (both described in [61]), JC–1 [62], LysoTracker Red DND–99, MitoTracker Red CMXRos (both described in [63]) and others) that are P-gp substrates and must be used in MDR cells with P-gp overexpression together with TQR or other suitable P-gp inhibitors.

MDC accumulated more in R and T cells after treatment with SFN than in S cells (Figure 8), and in R and T cells, we observed concentration-dependent CC arrest in the G0/G1 phase under the influence of SFN (Figure 6). In contrast to SFN, after treatment with AITC cells, MDC accumulated more in S and T cells than in R cells (Figure 8), and we also observed CC arrest in the G2/M phase in S and T cells after this treatment (Figure 6). Thus, this result could be considered a verification of the above-described idea that under the arrest of CC, autophagy was activated to obtain sufficient material and energy for the repair of damage induced by ITCs.

Similar to MDC, LTG can be used to detect autophagy [36]. In this case, we observed a shift in LTG fluorescence to higher intensities in cells treated with SFN or AITC, as measured by flow cytometry (Figure 9). However, this shift was not large enough to make reliable quantifications depending on the addition of SFN and AITC.

## 4. Materials and Methods

### 4.1. Chemicals

SFN (1-Isothiocyanato-4-(methanesulfinyl)butane) and AITC (3-Isothiocyanatoprop-1-ene) were obtained from Sigma-Aldrich (MERCK spol. s.r.o., Bratislava, Slovak Republic), unless otherwise stated in the text. All chemicals were from MERCK and were analytical grade.

### 4.2. Cell Culture and Cultivation Conditions

The murine cancer cell line of leukemic origin L1210 was obtained from Leibniz-Institut DSMZ-Deutsche Sammlung von Mikroorganismen und Zellkulturen GmbH (Braunschweig, Germany). We used three variants of the mouse lymphocytic leukemia cell line L1210: i. P-gp-negative drug-sensitive parental L1210 cells S (ACC–123. S) obtained from Leibniz-Institut DSMZ-Deutsche Sammlung von Mikroorganismen und Zellkulturen GmbH (Braunschweig, Germany); ii. P-gp-positive drug-resistant cells R overexpressing P-gp due to selection with vincristine [64]; iii. P-gp-positive drug-resistant T cells overexpressing P-gp due to stable transfection with Addgene plasmid 10957 (pHaMDRwt) and a retrovirus encoding full-length P-gp cDNA [65]. Both P-gp-positive variants R and T express high levels of P-gp at the mRNA and protein levels, show efflux activity in the calcein retention assay and are more than 100 times less sensitive to P-gp substrates (doxorubicin, vincristine) than S cells [23,32,66]. All of these characteristics are routinely checked in our variants of L1210 cells. Transfection and cell characterization were completed as described elsewhere [23]. Cells were incubated in RPMI 1640 media with l-glutamine (1 mg cm^−3^). Four percent fetal bovine serum and 1 μg cm^−3^ gentamycin (all purchased from Gibco, Langley, OK, USA) at 37 °C in a humidified atmosphere with 5% CO_2_. This procedure is termed passaging and was repeated three times per week. All cell variants (S. R and T) were cultivated in the absence or presence of the respective ITCs SFN or AITC at a concentration range of 2.5–30 µM and were used for further examination.

### 4.3. Counting of Viable S, R and T Cells after Passage in the Presence of ITCs

S, R and T cells (10^6^ cells/well) were cultured in medium containing SFN or AITC in a concentration range of 5–30 μM in 6-well culture plates (5 mL per well) for different time intervals (4–24 h). During this time interval, cells grew practically linearly. Cell viability was monitored by measuring the plasma membrane integrity of individual cells through changes in electrical resistance induced by cells that were passed through the detector in the CASY Model TT Cell Counter (Roche Applied Sciences, Madison, WI, USA) according to the manufacturer’s protocol. The number of viable cells as a function of ITC concentration and culturing time were fitted according to Equation by nonlinear regression using SigmaPlot 8.0 software (Systat Software. Inc., San Jose, CA, USA):

N=106+N0×t×exp[ln(0.5)×(cIC50)n]

where N is the number of viable cells after culturing time t in medium containing the respective ITC in concentration c; N_0_ is the number of viable cells after culturing time t in the absence of ITCs; IC_50_ is the median lethal concentration of the respective ITC; 10^6^ represents the initial cell inoculum given to each well.

Equation was derived from the previously described equation for dose-response dependence [25], considering the fact that the cell number increases linearly during 24 h culture.

### 4.4. Cell Metabolic Activity Estimation Using the MTS Assay

Cell metabolic activity was assessed in terms of the NADH- and NADPH-dependent reduction of MTS ([3-(4,5-Dimethylthiazol-2-yl)-5-(3-carboxymethoxyphenyl)-2-(4-sulfophenyl)-2*H*-tetrazolium inner salt]) to soluble formazan using the CellTiter 96^®^ AQueous One Solution Cell Proliferation Assay according to the manufacturer’s protocol (Promega, Madison, WI, USA, G3580). S. R and T cells were seeded in 6-well plates (5 mL per well) at a density of 3 × 10^5^ cells/mL. Cells treated with AITC were seeded in 60 mm Petri dishes to avoid the influence of evaporated AITC on neighboring wells in the well plate. The cells were exposed to SFN or AITC at different concentrations (5, 10, 15, 20, 25 and 30 μM), and the cell metabolic activity by MTS after 24 and 48 h was assessed. Then, 100 μL aliquots of cell suspension were incubated with MTS reagent (in dilution 1:6) for 3 h, and the absorbance of the soluble formazan product was recorded at 490 nm using a Universal Microplate Spectrophotometer μQuant (BioTek Instruments Inc., Winooski, VT, USA). Experiments were performed in triplicate, and the means of relative absorbance were used as a measure of cell metabolic activity.

### 4.5. Detection of Cell Death Mode Using Double Staining with FITC-Annexin V and Propidium Iodide

Cells were stained using an Annexin V-FLUOS Staining Kit (Roche. Mannheim. Germany) according to the manufacturer’s protocol. Briefly, S, R and T cells were seeded at a concentration of 3 × 10^5^/mL on dishes and treated with different concentrations of ITCs SFN or AITC for 48 h. A total of 5 × 10^5^ cells were collected and washed with phosphate buffered saline (PBS) and resuspended in 100 μL of binding buffer (10 mM HEPES/NaOH, pH 7.5 containing 140 mM NaCl and 2.5 mM CaCl_2_) at a concentration of approximately 5 × 10^6^ cells/mL. The cell suspension was added to plastic test tubes. Then, 0.25 μL of Annexin V-FITC (resulting concentration: 0.5 μg/mL) was added to each cell suspension. The mixtures were incubated in the dark for 15 min at room temperature. This was followed by the addition of 1 μL propidium iodide (final concentration 0.6 µg/mL) to each sample, which was immediately analyzed by flow cytometry on an Accuri C6 flow cytometer.

### 4.6. Cell Cycle Analysis

Cells (1 × 10^6^/mL) were incubated for 8 h and 12 h in the absence or presence of SFN (2.5, 5, 7.5 and 10 μM) and AITC (5, 10, 15, 20 μM), respectively. A total of 2 × 10^6^ cells were washed with cold PBS and fixed with 70% ethanol for 1 h. Cells were washed again with PBS and dissolved in 100 μL PBS containing 0.1 mg/mL RNase A (Thermo Fisher Scientific, Waltham, MA, USA) and incubated at 37 °C for 30 min in the dark. The final mixtures were cooled on ice for 10 min, and propidium iodide (40 µg/mL) was added to each sample; the samples were then incubated on ice for another 30 min. Finally, the specimens were evaluated by flow cytometry on an Accuri C6 flow cytometer.

### 4.7. RT-PCR Conditions

The cells were exposed to SFN at various concentrations (2.5, 5, 7.5 and 10 μM) or AITC (5, 10, 15, 20 and 30 μM). After the cultivation period (48 h), cells were harvested by centrifugation (664× *g* at 20 °C) and washed twice in PBS. Total RNA was isolated from cells using TRIREAGENT^®^ (MRC, Cincinnati, OH, USA) according to the manufacturer’s instructions. RNA samples were then quantified using a NanoDrop machine (λ260/280). Reverse transcription (RT) was performed with 1 μg of total RNA using Fermentas RevertAid™ Reverse Transcriptase for cDNA synthesis (Thermo Fisher Scientific, Waltham, MA, USA).

PCR was performed in a total volume of 25 μL using a PCR kit according to the manufacturer’s protocol (Thermo Fisher Scientific). The polymerase chain reaction (PCR) reaction mixture contained 1 μL of cDNA sample to screen for the expression of the following genes: *Bax*, *Bcl-2*, *Nfkb1*, *Nfkb2*, *RelA* and *Gapdh*. PCR was carried out using the following process: 30 cycles of 30 s denaturation at 95 °C; 40 s annealing at 58 °C for *Bax* and *Bcl-2*, 60 °C for *Nfkb1*, *Nfkb2*, *RelA* and *RelB*, 56.5 °C for *Gapdh*; and 2 min extension at 72 °C. The PCR products were separated on a 1.5% agarose gel (Lonza. Rockland, Rockland, ME, USA) and visualized using GelRed^TM^ nucleic acid gel stain (Biotium, Fremont, CA, USA). The primer sequences are documented in Table 2.

### 4.8. Western Blotting

Protein was extracted from cultured cells using RIPA lysis buffer containing 50 mM Tris-Cl (pH 8.0), 1% Triton X-100, 0.5% sodium deoxycholate, 0.1% SDS, 150 mM NaCl, and protease inhibitor cocktail from Sigma-Aldrich (Saint Louis, MO, USA). The proteins from the samples were separated by sodium dodecyl sulfate-polyacrylamide electrophoresis using 12% polyacrylamide (SDS-PAGE) gels using the protocol published by Laemmli [68]. The proteins were transferred by electroblotting onto nitrocellulose membranes (GE Healthcare Europe GmbH, Vienna, Austria) using the Towbin protocol [69,70]. The membranes were blocked for 1 h in 5% defatted milk solution in Tris-buffered saline-Tween 20 (TBST). The primary antibodies against LC3B (L7543, Sigma-Aldrich, Saint Louis, MO, USA) diluted 1:300, Bax (N-20), Bcl2 (N-19), NFκ-B p52 (K-27), NFκ-B p50 (H-119), NFκ-B p65 (C-20), all from Santa Cruz Biotechnology, Dallas, TX, USA, diluted 1:100 and GAPDH (MAB374, EMD Millipore Chemicals, Billerica, MA, USA), were added to the blocking buffer containing 5% defatted milk overnight at 4 °C. IgG kappa binding protein (*m*-IgGκ BP) and mouse anti-rabbit IgG-HRP both conjugated with horseradish peroxidase (HRP) were used at a dilution of 1:1000 as secondary antibodies and were purchased from Santa Cruz Biotechnology. HRP signals were visualized using an ECL detection system (GE Healthcare Europe GmbH, Vienna, Austria) on an Amersham Imager 600 (GE Healthcare Europe GmbH, Pittsburgh, PA, USA).

### 4.9. Visualization of Autophagic Vacuoles by MDC

The autofluorescent lysosomotropic compound MDC was recently introduced as a specific autophagolysosome marker to analyze the autophagic process [59]. S, R and T cells (10^5^) treated or untreated with either SFN (5, 10 and 20 μM) or AITC (10, 20 and 30 μM) were resuspended in 200 μL of PBS containing 50 μM MDC and 500 nM TQR (SelleckChem, Houston, TX, USA), and cells were incubated at 37 °C for 45 min in the dark. After incubation, the cells were washed two times with PBS. To label cell surface wheat germ agglutinin (WGA) conjugated with Texas Red (Invitrogen, ThermoFisher Scientific, Eugene, OR, USA) at a final concentration of 10 μM was added to 200 µL of PBS. MDC was visualized with a 5% UV diode at 405 nm and emission spectra in the range 515 nm–574 nm, whether (WGA) conjugated with Texas Red was stimulated with a 7% white laser at 58.9 nm and emission spectra in the range 599 nm–661 nm. Imaging was performed on a Leica TCS SP8 AOBS confocal microscope (Leica Microsystems, Germany) with an objective HC PL APO CS2 63 × /1.40 OIL.

### 4.10. Staining of S, R and T Cells after Treatment with either SFN or AITC by LTG

S, R and T cells (10^5^) treated and untreated with either SFN or AITC (2.5 and 20 μM) were resuspended in 200 μL of RPMI medium without fetal bovine serum. TQR was added to this mixture at a final concentration of 500 nM, and then, lysosome-specific dye LysoTracker^®^ green DND-26 (LTG, Thermo Fisher Scientific s.r.o., Carlsbad, CA, USA) was added to a final concentration of 75 nM. According to the manufacturer’s instructions, cells were incubated with dye in the dark at 37 °C for 1 h. Cells were then washed in PBS buffer and resuspended in 200 µL of PBS. Afterwards, cells were counted in a BD Accuri C6 flow cytometer (BD Bioscience, San Jose, CA, USA) according to the protocol given in the Accuri cytometer’s application note.

## 5. Conclusions

Both SFN and AITC are capable of inducing cellular damage leading to cell death in P-glycoprotein-negative S cells and in P-gp-positive R and T cells. However, used ITCs act differently. SFN causes more intensive cell death (probably necrotic) in S cells. In R and T cells, we observed cell cycle arrest in the G0/G1 phase. We also observed more intensive labeling with MDC in R and T cells than in S cells after treatment with SFN, suggesting a higher course of autophagy. When measuring metabolic activity with the MTS assay, we observed a significant increase in signal at lower SFN concentrations in R and T cells, suggesting the hyperactivation of the metabolism. AITC causes the same hyperactivation of the metabolism in R and T cells to a lesser extent. In addition, AITC causes a more pronounced cell death effect in R cells and, at higher concentrations, in T cells than in S cells. AITC induces cell cycle arrest in the G2/M phase in S and T cells, and in these cell variants, AITC also increases labeling with MDCs.

These differences in the effect of both ITCs are due to their different physicochemical properties.

## Figures and Tables

**Table 1:** Values of IC_50_ for SFN or AITC obtained by nonlinear regression of data for S, R and T cell proliferation according to Equation.

Variant of L1210 Cells	SFN IC_50_	AITC IC_50_
	(μM)	(μM)
S	10.30 ± 0.45	6.80 ± 1.68
R	8.98 ± 0.55	4.79 ± 1.16
T	12.10 ± 0.63	10.05 ± 1.59

**Table 2:** PCR primer structures.

Gene	Primer Sequences	T_A_ (°C)	PCR Product (bp)
*Bax ^a^*	F: 5′-CTAGCAAAGTAGAAGAGGGCAACC-3′	58	151
	R: 5′-ATGAACTGGACAGCAATATGGAG-3′		
*Bcl-2 ^a^*	F: 5′-GCATGCTGGGGCCATATAGTT-3′	58	323
	R: 5′-GGCTGGGGATGACTTCTCTC-3′		
*Nfkb1 ^b^*	F: 5′-GAAATTCCTGATCCAGACAAAAAC-3′	60	194
	R: 5′-ATCACTTCAATGGCCTCTGTGTAG-3′		
*Nfkb2 ^b^*	F: 5′-CTGGTGGACACATACAGGAAGAC-3′	60	195
	R: 5′-ATAGGCACTGTCTTCTTTCACCTC-3′		
*RelA ^b^*	F: 5′-CTTCCTCAGCCATGGTACCTCT-3′	60	167
	R: 5′-CAAGTCTTCATCAGCATCAAACTG-3′		
*RelB ^b^*	F: 5′-CTTTGCCTATGATCCTTCTGC-3′	60	150
	R: 5′-GAGTCCAGTGATAGGGGCTCT-3′		
*Gapdh ^a^*	F: 5′-CAATGTGTCCGTCGTGGAT-3′	56.5	314
	R: 5′-GTGGGTGGTCCAGGGTTT-3′		

^a^ Primers designed by program Primer 3 using the databases NCBI and Ensemblelibrary. ^b^ Primers adopted from [67].

## Supplementary Materials

The following are available online, Figure S1: FACS dot plots of cell death induced in S, R and T cells by SFN and AITC; Figure S2: RT-PCR detection of *Bax*, *Bcl-2*, *Nfkb1*, *RelA*, *Nfkb2*, and *RelB* transcripts in S, R and T cells; Figure S3: FACS histograms of cell cycle detection by PI staining; Figure S4: Visualization of autophagic vesicles in S and R cells using MDC; Table S1. Mode of cell death after treatment of S, R and T cells for 48 h in medium containing 30 μM AITC.

## Abbreviations

AITC	allyl isothiocyanate
CC	cell cycle
FAV	fluorescein isothiocyanate labeled annexin-V
HRP	Horseradish Peroxidase
ITCs/ITC	isothiocyanates/isothiocyanate
LTG	LysoTracker^®^ green DND-26
MDR	multidrug resistance
MDC	monodansyl cadaverine
MTS	[3-(4,5-Dimethylthiazol-2-yl)-5-(3-carboxymethoxyphenyl)-2-(4-sulfophenyl)-2H-tetrazolium inner salt]
PBS	phosphate buffered saline
P-gp	P-glycoprotein
PI	propidium iodide
R	drug-resistant P-gp-positive L1210 cells obtained by selection with vincristine
S	drug sensitive parental P-gp-negative L1210 cells
SDS-PAGE	Sodium dodecyl sulfate-polyacrylamide gel electrophoresis
SFN	sulforaphane
T	drug-resistant P-gp-positive L1210 cells obtained by transfection with the human P-gp gene
TBST	tris-buffered saline-Tween 20
TQR	tariquidar
WGA	wheat germ agglutinin
