# Supplementary material for: Cell Death Effects Induced by Sulforaphane and Allyl Isothiocyanate on P-Glycoprotein Positive and Negative Variants in L1210 Cells
Source: Molecules. 2020 Apr 30;25(9):2093. doi: 10.3390/molecules25092093 (PMC7249010; doi:10.3390/molecules25092093)
Supplement: Supplementary file 1 [file molecules-25-02093-s001.pdf]

Supplementary Material

Cell Death Effects Induced by Sulforaphane and Allyl Isothiocyanate on P-glycoprotein Positive and Negative Variants in L1210 Cells.

Szilvia Kontar [1], Denisa Imrichova [1,2], Anna Bertova [1], Katarina Mackova [1], Alexandra Poturnayova [1], Zdena Sulova [1], Albert Breier[1,2,]

[1] Institute of Molecular Physiology and Genetics, Centre of Biosciences, Slovak Academy of Sciences, Dúbravská cesta 9, 84005 Bratislava, Slovakia

[2] Institute of Biochemistry and Microbiology, Faculty of Chemical and Food Technology, Slovak University of Technology in Bratislava, Radlinského 9, 81237 Bratislava, Slovakia

Table S1. Mode of cell death after treatment of S, R and T cells for 48 h in medium containing 30 µM AITC

**Variant of L1210 cells**	**Viable FAV$^-$, PI$^-$**	**Apoptotic FAV$^+$, PI$^-$**	**Necrotic FAV$^-$, PI$^+$**	**Late Stage** FAV$^+$, PI$^+$
S	76±8	2±2	2±2	20±4
R	3±3*	2±2	9±7	86±9*
T	3±3*	1±1	11±8	83±8*

* differs from S at the p<0.01 level

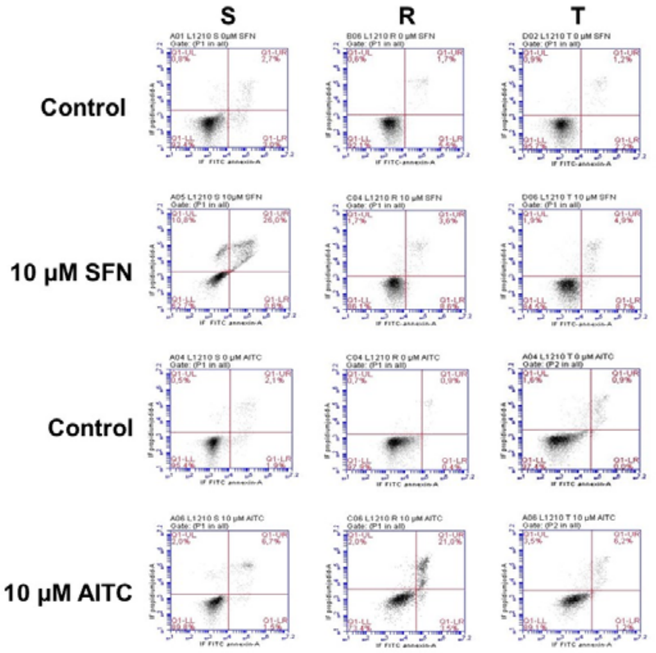

Figure S1. FACS dot plots of cell death induced in S, R and T cells by SFN and AITC using apoptosis and necrosis detection by FAV/PI double staining. The cells were incubated for 48 h in the absence (control) or presence of 10 µM of either SFN or AITC prior to the measurements. The dot blots are representative of three independent experiments.

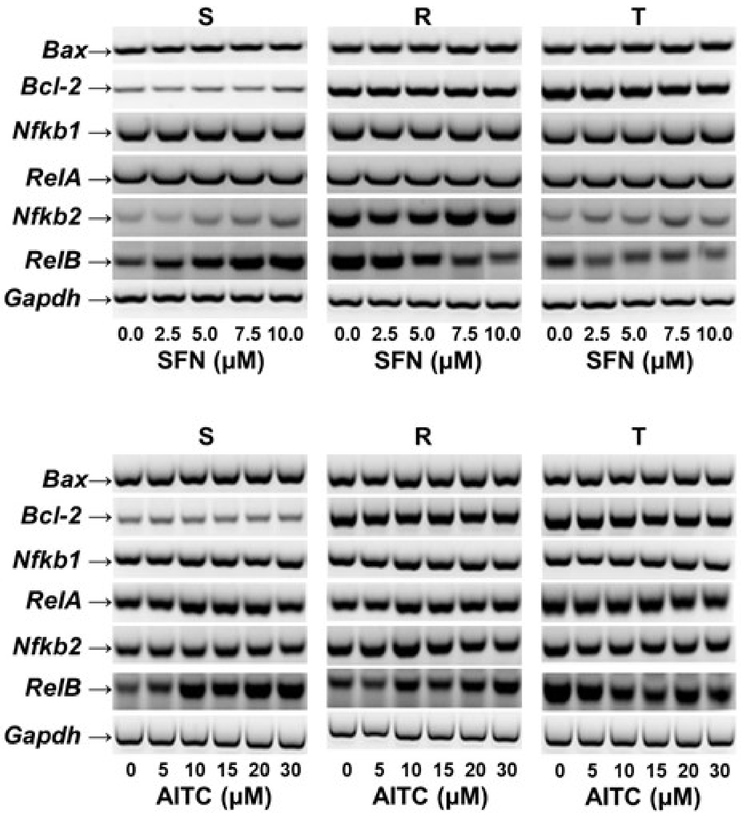

Figure S2. RT-PCR detection of *Bax, Bcl-2, Nfkb1, RelA, Nfkb2,* and *RelB transcripts* in S, R and T cells after 24 h incubation in medium containing SFN or AITC at given concentrations. *Gapdh* was used as an internal control. Data are representative of three independent measurements.

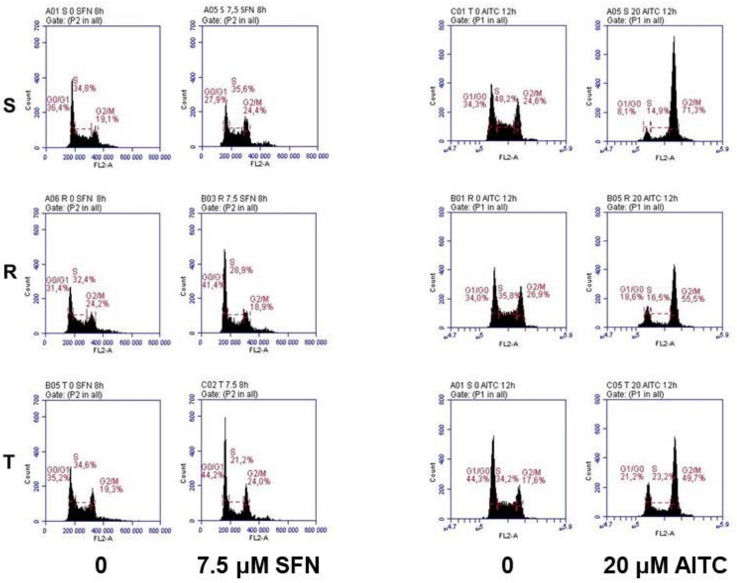

Figure S3. FACS histograms of cell cycle detection by PI staining. S, R and T cells after cultivation for given time in medium containing either SFN (at concentrations of 0.0 and 7.5 μM) or AITC (at concentrations of 0 and 20 μM) were used for detection. The histograms are representative of three independent experiments.

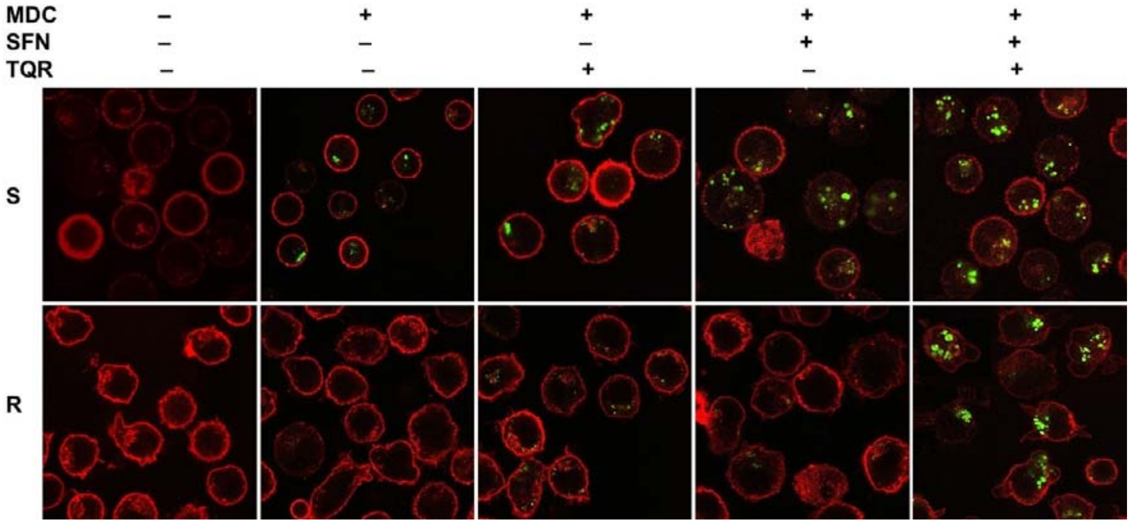

Figure S4. Visualization of autophagic vesicles in S and R cells using MDC. Cells (S and R) were stained after 24 h of treatment in the absence or presence of SFN (10 µM) with MDC. Staining with MDC was performed in the absence or presence of 500 nM TQR. Data are representative of three independent experiments.
